# Secretory carrier-associated membrane protein 2 (SCAMP2) regulates cell surface expression of T-type calcium channels

**DOI:** 10.1186/s13041-021-00891-7

**Published:** 2022-01-03

**Authors:** Leos Cmarko, Robin N. Stringer, Bohumila Jurkovicova-Tarabova, Tomas Vacik, Lubica Lacinova, Norbert Weiss

**Affiliations:** 1 Institute of Biology and Medical Genetics, First Faculty of Medicine, Charles University, Prague, Czech Republic; 2 Institute of Organic Chemistry and Biochemistry, Czech Academy of Sciences, Prague, Czech Republic; 3 Department of Pathophysiology, Third Faculty of Medicine, Charles University, Prague, Czech Republic; 4 Center of Biosciences, Institute of Molecular Physiology and Genetics, Slovak Academy of Sciences, Bratislava, Slovakia

**Keywords:** Ion channels, Calcium channels, T-type channels, Ca_v_3.2 channels, Secretory carrier-associated membrane protein 2, SCAMP2, Trafficking

## Abstract

Low-voltage-activated T-type Ca^2+^ channels are key regulators of neuronal excitability both in the central and peripheral nervous systems. Therefore, their recruitment at the plasma membrane is critical in determining firing activity patterns of nerve cells. In this study, we report the importance of secretory carrier-associated membrane proteins (SCAMPs) in the trafficking regulation of T-type channels. We identified SCAMP2 as a novel Ca_v_3.2-interacting protein. In addition, we show that co-expression of SCAMP2 in mammalian cells expressing recombinant Ca_v_3.2 channels caused an almost complete drop of the whole cell T-type current, an effect partly reversed by single amino acid mutations within the conserved cytoplasmic E peptide of SCAMP2. SCAMP2-induced downregulation of T-type currents was also observed in cells expressing Ca_v_3.1 and Ca_v_3.3 channel isoforms. Finally, we show that SCAMP2-mediated knockdown of the T-type conductance is caused by the lack of Ca_v_3.2 expression at the cell surface as evidenced by the concomitant loss of intramembrane charge movement without decrease of total Ca_v_3.2 protein level. Taken together, our results indicate that SCAMP2 plays an important role in the trafficking of Ca_v_3.2 channels at the plasma membrane.

**Supplementary Information:**

The online version contains supplementary material available at 10.1186/s13041-021-00891-7.

Through their ability to pass calcium ions (Ca^2+^) near the resting membrane potential, low-voltage-activated T-type channels have an important physiological role in shaping firing activity patterns of nerve cells, both in the central and peripheral nervous system. The implication of T-type channels in the control of neuronal excitability is partly defined by the density of channels embedded in the plasma membrane. Therefore, a number of molecular mechanisms and signaling pathways come into play to underly precise control of cell surface expression of T-type channels [1] and defects whether genetic or acquired can lead to severe neuronal conditions [2, 3].

Secretory carrier-associated membrane proteins (SCAMPs) form a family of integral membrane proteins essentially expressed in the trans-Golgi network and recycling endosome membranes where they regulate vesicular trafficking and vesicle recycling processes [4]. Of the five known mammalian SCAMPs, SCAMP2 shows a ubiquitous expression pattern including in neuronal tissues where SCAMP2 transcripts are observed for instance in the cerebellum, thalamus, hippocampus, and spinal cord (https://www.proteinatlas.org/ENSG00000140497-SCAMP2/tissue). SCAMP2 consists of four transmembrane helices with cytoplasmic amino- and carboxy-termini and a so-called E peptide located between transmembrane helices 2 and 3 essential for mediating SCAMP2 function [5]. This E domain is highly conserved among SCAMP isoforms and represents an essential molecular determinant for SCAMP2-mediated inhibition of exocytosis [6]. Only a few reports have documented the role of SCAMP2 in the regulation of ion channels and transporters [7–10]. In the present study, we aimed to assess the functional role of SCAMP2 in the regulation of T-type channels.

To address this issue, we assessed whether Ca_v_3.2 channels and SCAMP2 associate at the protein level. Co-immuniprecipitation from tsA-201 cells expressing recombinant HA-tagged Ca_v_3.2 and Myc-tagged SCAMP2 using an anti-HA-antibody precipitated SCAMP2-Myc with Ca_v_3.2-HA revealing the existence of a Ca_v_3.2/SCAMP2 protein complex (Fig. 1a). We note that co-immunoprecipitation experiments from total cell lysates do not address whether this interaction is direct or not and it is a possibility that formation of Ca_v_3.2/SCAMP2 protein complex may also involve another intermediate protein. Next, we aimed to analyze the functional effect of SCAMP2 on Ca_v_3.2 channels. Patch-clamp recordings from tsA-201 cells expressing Ca_v_3.2 showed that co-expression of SCAMP2 produces an almost complete drop of the whole-cell T-type current (Fig. 1b and c). For instance, the maximal macroscopic conductance (*G*_max_) was reduced by 91% (*p* < 0.0001) in cells co-expressing SCAMP2 (61 ± 18 pS/pF, n = 18) compared to cells expressing Ca_v_3.2 alone (692 ± 62 pS/pF, n = 25) (Fig. 1d). Alanine mutagenesis of the E peptide of SCAMP2 at cysteine 201 (C201A) and tryptophan 202 (W202A) reduced this effect to 64% (*p* = 0.0269) and 39% (*p* < 0.0001) inhibition, respectively, indicating that SCAMP2-induced knockdown of Ca_v_3.2 currents is at least partly mediated by the E peptide (Fig. 1b–d). These data also indicate that the reduction in Ca_v_3.2 current density in the presence of SCAMP2 is not merely due to the co-expression of just any protein given that the W202A mutant construct has no big effect. With regard to the effect of SCAMP2 on the other T-type channel isoforms, co-expression of SCAMP2 in cells expressing recombinant Ca_v_3.1 and Ca_v_3.3 reduced *G*_max_ by 35% (p < 0.0001) and 98% (p < 0.0001) respectively (Fig. 1e and f and Additional file 1: Fig. S1) indicative of a differential susceptibility to SCAMP2-dependent modulation (Ca_v_3.3 ≈ Ca_v_3.2 > Ca_v_3.1). Next, we aimed to assess the underlying mechanism by which SCAMP2 induced knockdown of the T-type conductance. The alteration of the T-type conductance in the presence of SCAMP2 could originate from an overall decreased level of Ca_v_3.2 proteins or from a reduced expression of the channel in the plasma membrane. Western blot analysis from total cell lysates showed that Ca_v_3.2 protein levels were not decreased by the presence of SCAMP2. Instead, we observed a non-significant trend toward higher expression levels which may have arisen from a lower rate of vesicular exocytosis therefore preventing the channel from being targeted to the proteasomal degradation machinery (Fig. **1**g and h). In contrast, recording of intramembrane charge movements (*Q*) that provide an accurate assessment of the number of channels embedded in the plasma membrane revealed an 85% decrease (p < 0.0001) of *Q*_max_ in cells expressing SCAMP2 (from 6.1 ± 0.7 fC/pF, n = 16 to 0.9 ± 0.2 fC/pF, n = 17) (Fig. 1i and j) indicating a decreased channel expression at the cell surface. Moreover, while the kinetics of intramembrane charge movements remained unaltered (Fig. 1k), the *G*_max_/*Q*_max_ dependency in the presence of SCAMP2 was reduced by 52% (p < 0.0001) (from 0.169 ± 0.007 pS/fC, n = 16 to 0.080 ± 0.014 pS/fC, n = 11) suggesting an additional alteration of the coupling between the activation of the voltage-sensor and the pore opening of the channel (Fig. 1l). This observation is consistent with a previous report showing that besides to be concentrated primarily in intracellular membranes, SCAMP2 is also found in the plasma membrane [11] and therefore could potentially modulate the gating of the channel in addition to its insertion in the membrane. We note that the reduction of *Q*_max_ combined with the reduction of *G*_max_/*Q*_max_ of the small fraction of channels that still reached the plasma membrane in the presence of SCAMP2 is very similar to the reduction of the maximal T-type conductance we previously observed (91%, Fig. 1d).

**Fig. 1:**
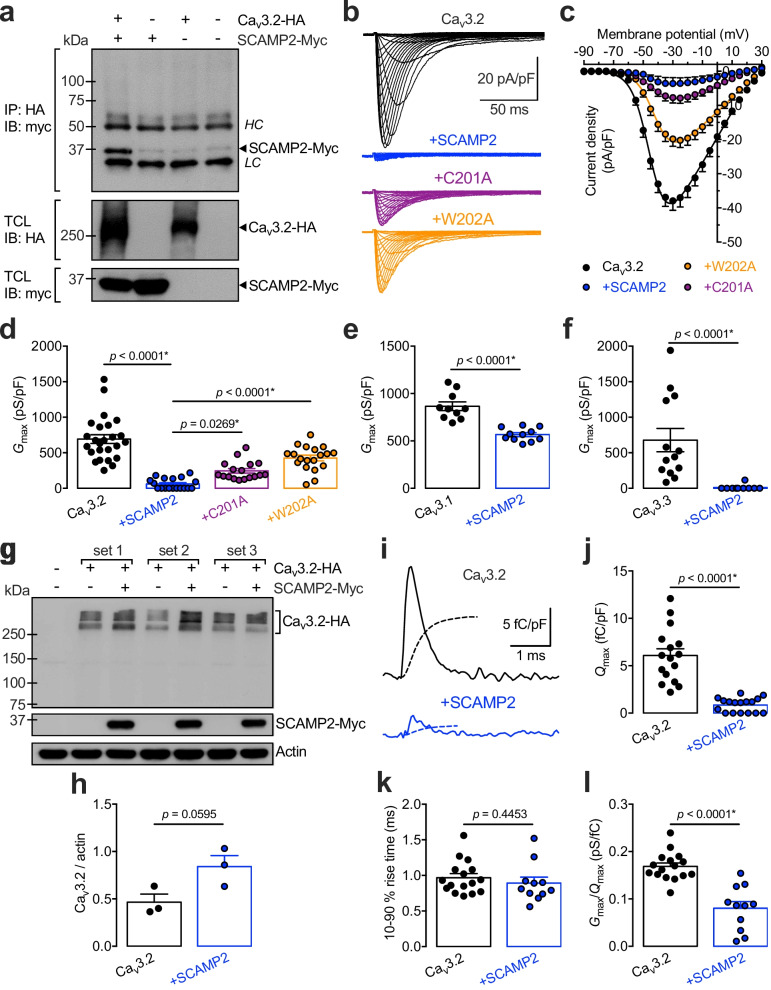
SCAMP2 regulates T-type channel expression. **a** Co-immunoprecipitation of Myc-tagged SCAMP2 (SCAMP2-Myc) from tsA-201 cells co-transfected with HA-tagged Ca_v_3.2 channel (Ca_v_3.2-HA). The upper panel shows the result of the co-immunoprecipitation of SCAMP2-Myc with Ca_v_3.2-HA using an anti-HA antibody. The lower panels show the immunoblot of Cav3.2-HA and SCAMP2-Myc from total cell lysates using an anti-HA and anti-Myc antibody, respectively. *HC*, heavy chain antibody; *LC*, light chain antibody. This experiment was performed four times from independent transfections and Ca_v_3.2/SCAMP2 interaction was consistently observed. **b** Representative T-type current traces from tsA-201 cells expressing Ca_v_3.2 alone (black traces) and in combination with wild-type SCAMP2 (blue traces), as well as with C201A (purple traces) and W202A (orange traces) SCAMP2 mutants in response to 150 ms depolarizing steps varied from − 90 mV to + 30 mV from a holding potential of − 100 mV. **c** Corresponding mean current/voltage (*I/V*) relationships. **d** Corresponding mean maximal macroscopic conductance values (*G*_max_) obtained from the fit of the *I*/*V* curves with the modified Boltzmann Eq. (1). **e–f** Mean *G*_max_ values for tsA-201 cells expressing Ca_v_3.1 and Ca_v_3.3 channels, respectively. **g**. Immunoblot of Ca_v_3.2-HA expressed in tsA-201 cells in the absence (−) and presence (+) of SCAMP2-Myc. The immunoblot shows the results of three independent sets of transfections. **h** Corresponding mean expression levels of Ca_v_3.2-HA normalized to actin. **i** Representative intramembrane charge movement traces recorded at the ionic reversal potential from cells expressing Ca_v_3.2 alone (black trace) and in the presence of SCAMP2 (blue trace). The doted lines depict the time course of the intramembrane charge mouvement integral. **j** Corresponding mean maximal intramembrane charge movement values (*Q*_max_). **k** Corresponding mean 10–90% rise time values calculated from the integral time course shown in **i**. **l** Corresponding mean *G*_max_/*Q*_max_ values

Several Ca_v_3.2 interacting proteins including KLHL1 [12], USP5 [13], Stac1 [14], calnexin [15], and Rack-1 [16] have been reported to modulate the sorting and trafficking of the channel to the plasma membrane. In this study, we reported SCAMP2 as a novel Ca_v_3.2-interacting partner and potent repressor of the expression of the channel at the cell surface. Further investigations will be necessary to fully explore the importance of this regulation in native conditions. Importantly, altered expression of SCAMP2 has been reported in several types of cancer [17]. Given the importance of Ca_v_3.2 channels in the development of peripheral painful neuropathies [18], it will be interesting to assess to what extent SCAMP2-mediated regulation of Ca_v_3.2 could possibly contribute to cancer-related neuropathic pain.

## Supplementary Information

**Additional file 1.** **Fig. S1**. Functional effect of SCAMP2 on Ca_v_3.1 and Ca_v_3.3 channels. **a** Representative T-type current traces from tsA-201 cells expressing Ca_v_3.1 alone (black traces) and in combination with SCAMP2 (blue traces) in response to 150 ms depolarizing steps varied from -90 mV to +30 mV from a holding potential of -100 mV. **b** Corresponding mean current/voltage (*I/V*) relationships. **c** Corresponding mean maximal macroscopic conductance values (G*max*) obtained from the fit of the *I/V* curves with the modified Boltzmann Eq. (1). **d**–**e** Same legend as for **a**–**c** but for cells expressing Ca_v_3.3 channel.

## Data Availability

All data generated or analyzed during this study are included in this published article and its additional information files.

## Abbreviations

*G*_max_: Maximal macroscopic conductance
KLHL1: Kelch-like 1
*Q*_max_: Maximal intra membrane charge movement
Rack-1: Receptor for activated C kinase 1
SCAMP2: Secretory carrier membrane protein 2
Stac1: Stac adaptor protein 1
USP5: Ubiquitin-specific proteinase 5
