# Supplementary material for: Secretory carrier-associated membrane protein 2 (SCAMP2) regulates cell surface expression of T-type calcium channels
Source: Mol Brain. 2022 Jan 3;15:1. doi: 10.1186/s13041-021-00891-7 (PMC8721997; doi:10.1186/s13041-021-00891-7)
Supplement: Supplementary file 1 — Additional file 1. Fig. S1. Functional effect of SCAMP2 on Cav3.1 and Cav3.3 channels. a Representative T-type current traces from tsA-201 cells expressing Cav3.1 alone (black traces) and in combination with SCAMP2 (blue traces) in response to 150 ms depolarizing steps varied from -90 mV to +30 mV from a holding potential of -100 mV. b Corresponding mean current/voltage (I/V) relationships. c Corresponding mean maximal macroscopic conductance values (Gmax) obtained from the fit of the I/V curves with the modified Boltzmann Eq. (1). d–e Same legend as for a–c but for cells expressing Cav3.3 channel. [file 13041_2021_891_MOESM1_ESM.docx]

**Secretory carrier-associated membrane protein 2 (SCAMP2) regulates cell surface expression of T-type calcium channels**

**Additional information**

**Additional methods**

**Plasmids and site-directed mutagenesis**

The human wild-type Myc-tagged SCAMP2 in pCMV3 was purchased from SinoBiological and was used as template to generate the C201A mutant by PCR using Q5 Site-Directed Mutagenesis Kit (NEB) and the following mutagenic primers: forward: 5´- TGCCTTCCTTGCTTGGTACCGACC-3´; reverse: 5´- CAGGGAGTGAAGATCAGAAAC-3´. The final construct was verified by sequencing of the coding region of the plasmid cDNAs. The human SCAMP2 W202A mutant was obtained from Dr. Jana Haase and was previously described [1]. The human HA-tagged Ca_v_3.2 (UniProt O95180-1) in pcDNA3.1 was previously described [2].

***Cell culture and heterologous expression***

Human embryonic kidney tsA-201 cells were grown in DMEM medium supplemented with 10% fetal bovine serum and 1% penicillin/streptomycin (all media purchased from Invitrogen) and maintained under standard conditions at 37^o^C in a humidified atmosphere containing 5% CO_2_. Heterologous expression was performed by transfecting cells with plasmid cDNAs encoding Ca_v_3.2-HA and SCAMP2-Myc in a 1:1 ratio and empty pEGFP vector as transfection marker. For cells transfected with Ca_v_3.2-HA alone, empty pcDNA3.1 plasmid was used as control.

***Co-immunoprecipitation***

For co-immunoprecipitation, tsA-201 cells expressing Ca_v_3.2-HA with SCAMP2-Myc were solubilized in lysis buffer (50 mM Tris/HCl, 150 mM NaCl, 1% Nonidet P-40; pH 7.5) supplemented with protease inhibitors cocktail (Sigma). Cell lysates were cleared by centrifugation at 15000 g for 20 min. All steps were carried out at 4^0^C. Lysates were incubated over-night at 4^0^C with a rat monoclonal anti-HA antibody (Roche) and then for 2h at 4^0^C with magnetic protein G beads (ThermoFisher). Beads were washed 3 times, resuspended in Laemmli buffer and heated at 100^0^C for 5 min.

**SDS-PAGE and immunoblot analysis**

Immunoprecipitation samples or total cell lysates were separated on a 10 % gradient SDS-PAGE and transferred onto PVDF membrane (Millipore). For detection of SCAMP2-Myc the membrane was incubated with a primary mouse monoclonal anti-Myc antibody (ThermoFisher) diluted at 1:2000. For detection of Ca_v_3.2-HA the membrane was incubated with a primary rat monoclonal anti-HA antibody (Roche) diluted at 1:2000. For detection of actin, the membrane was incubated with a primary mouse monoclonal anti-actin antibody (Sigma). Membranes were then washed in PBS/Tween-20 buffer and incubated with a secondary anti-mouse or anti-rat HRP-conjugated antibody (Jackson ImmunoResearch), respectively, diluted at 1:10 000. Immunoreactive bands were detected by enhanced chemiluminescence and analyzed using ImageJ software.

***Electrophysiology***

Patch clamp recording of T-type currents was performed 72 h after transfection in the whole-cell configuration at room temperature (22–24°C). The bath solution contained (in millimolar): 5 BaCl2, 5 KCl, 1 MgCl2, 128 NaCl, 10 TEA-Cl, 10 D-glucose, 10 4-(2-hydroxyethyl)-1-piperazineethanesulfonic acid (HEPES) (pH 7.2 with NaOH). Patch pipettes were filled with a solution containing (in millimolar): 110 CsCl, 3 Mg-ATP, 0.5 Na-GTP, 2.5 gCl2, 5 D-glucose, 10 EGTA, and 10 HEPES (pH 7.4 with CsOH), and had a resistance of 2–4 MΩ. Recordings were performed using an Axopatch 200B amplifier (Axon Instruments) and acquisition and analysis were performed using pClamp 10 and Clampfit 10 software, respectively (Axon Instruments). The linear leak component of the current was corrected online, and current traces were digitized at 10 kHz and filtered at 2 kHz. The voltage dependence of activation of Ca_v_3.2 channels was determined by measuring the peak T-type current amplitude in response to 150 ms depolarizing steps to various potentials applied every 10 s from a holding membrane potential of −100 mV. The current-voltage relationship (*I*/*V*) curve was fitted with the following modified Boltzmann Equation (1):

$$\left( 1 \right) I\left( V \right)= Gmax \frac{(V-Vrev)}{1+ \exp\frac{(V0.5-V)}{k}}$$

with *I*(*V*) being the peak current amplitude at the command potential *V*, *G*max the maximum conductance, *V*rev the reversal potential, *V*_0.5_ the half-activation potential, and *k* the slope factor.

Recording of intramembrane charge movement was performed in a bath solution containing (in millimolar): CsCl 95; TEACl 40, BaCl2 5; MgCl2 1; HEPES 10; glucose 10; pH 7.4 (adjusted with CsOH). Patch pipettes had a resistance ranging from 1.8 MΩ to 2.2 MΩ when filled with a solution containing (in millimolar): CH3SO3Cs 130; Na-ATP 5; TEACl 10; HEPES 10; EGTA 10; MgCl2 5; pH 7.4 (adjusted with CsOH). Osmolarity of the intracellular solution was approximately 300 mOsmol/L. Osmolarity of the extracellular solution was adjusted by adding sucrose so that the final value was about 2–3 mOsmol/L lower than the osmolarity of the corresponding intracellular solution. Recordings were performed using a HEKA EPC10 amplifier (HEKA Electronics). Acquisition and analysis were performed using Patchmaster v90.2 and Fitmaster v2x73.1 and Origin Pro 2015 software, respectively. Only cells with an input resistance less than 5 MΩ were considered. The input resistance and capacity transients were compensated by up to 70% with in-built circuits of the EPC 10 amplifier. Remaining artifacts were subtracted using a P/8 procedure. ON-gating currents (Q_ON_) were recorded in response to a series of 5 depolarizing pulses at the reversal potential of the ionic current assessed for each cell, and total gating charge Qmax was calculated as the integral of the area below the averaged current traces. The time course of Q_ON_ integral was used to determine 10-90% rise time of intramembrane charge movement.

***Statistical analysis***

Data values are presented as mean ± S.E.M for *n* measurements. Statistical analysis was performed using GraphPad Prism 7. For datasets passing the D’Agostino & Person omnibus normality test, statistical significance was determine using a Student’s t-test. For multiple comparison analyses, one-way analysis of variance (ANOVA) followed by Dunnett’s post hoc multiple comparisons test was used. Datasets were considered significantly different for *p* < 0.05 *.

**Additional figures**


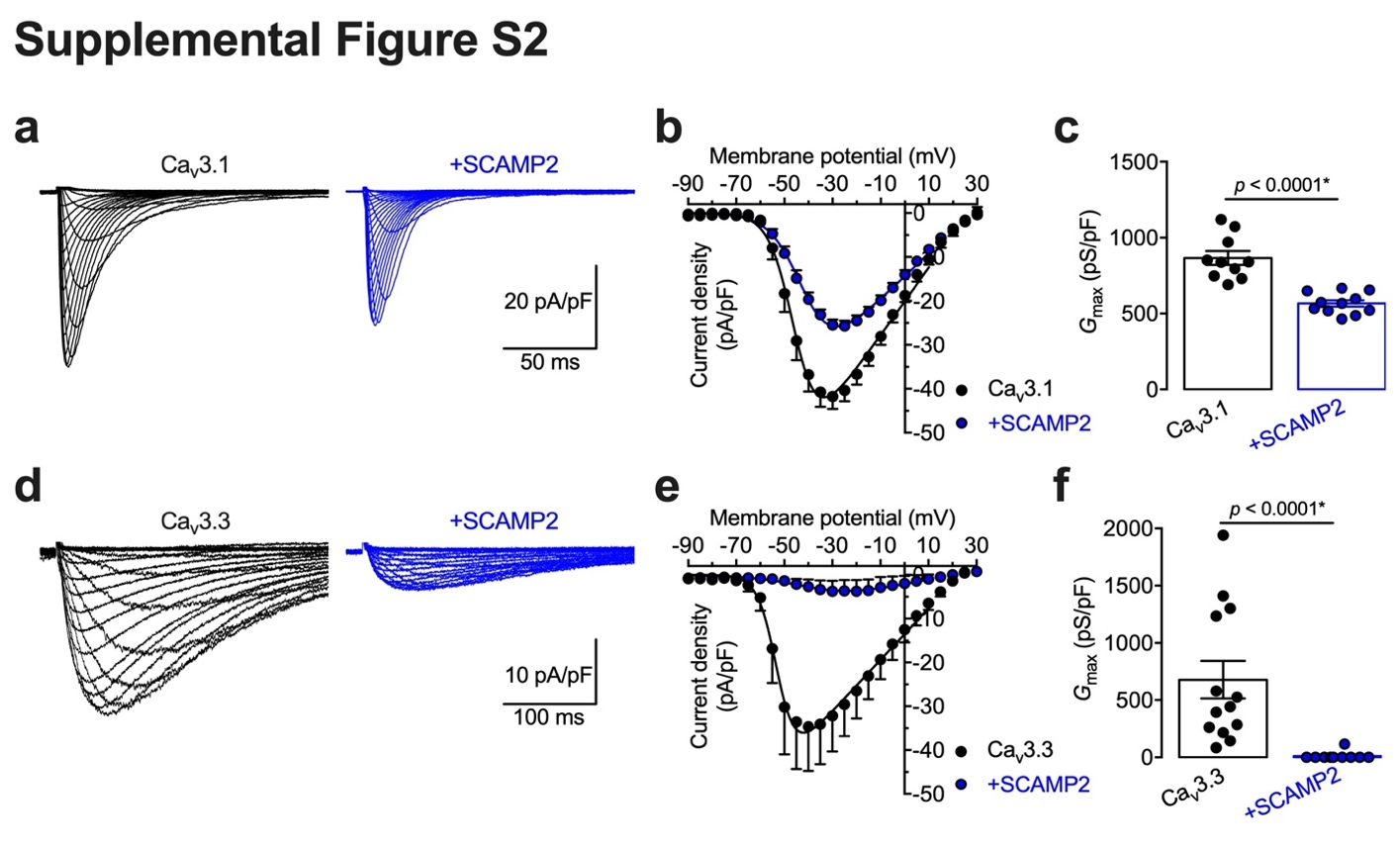


**Fig. S1** Functional effect of SCAMP2 on Ca_v_3.1 and Ca_v_3.3 channels. **a**. Representative T-type current traces from tsA-201 cells expressing Ca_v_3.1 alone (black traces) and in combination with SCAMP2 (blue traces) in response to 150 ms depolarizing steps varied from -90 mV to +30 mV from a holding potential of -100 mV. **b**. Corresponding mean current/voltage (*I/V*) relationships. **c**. Corresponding mean maximal macroscopic conductance values (*G*_max_) obtained from the fit of the *I*/*V* curves with the modified Boltzmann Eq. (1). **d-e** Same legend as for **a-c** but for cells expressing Ca_v_3.3 channel.

**Additional references**

1. Müller HK, Wiborg O, Haase J. Subcellular redistribution of the serotonin transporter by secretory carrier membrane protein 2. J Biol Chem. 2006;281(39):28901–9.

2. Dubel SJ, Altier C, Chaumont S, Lory P, Bourinet E, Nargeot J. Plasma membrane expression of T-type calcium channel alpha(1) subunits is modulated by high voltage-activated auxiliary subunits. J Biol Chem. 2004;279(28):29263–9.
